# Investigating myotoxicity following Australian red-bellied black snake (*Pseudechis porphyriacus*) envenomation

**DOI:** 10.1371/journal.pone.0256653

**Published:** 2021-09-10

**Authors:** Suchaya Sanhajariya, Stephen B. Duffull, Geoffrey K. Isbister

**Affiliations:** 1 Clinical Toxicology Research Group, University of Newcastle, Newcastle, New South Wales, Australia; 2 School of Pharmacy, University of Otago, Dunedin, New Zealand; Universidad de Costa Rica, COSTA RICA

## Abstract

**Background:**

Myotoxicity is one of the common clinical manifestations of red-bellied black snake (*Pseudechis porphyriacus*) envenomation characterised by elevated creatine kinase (CK) concentrations of greater than 1000 U/L. This study aimed to investigate the occurrence of myotoxicity in patients following envenomation.

**Methods/Principal findings:**

Patient characteristics and serial blood samples (timed venom concentrations and CK concentrations, pre- and post- antivenom) from 114 patients (median age 41, 2-90y; 80 male) were extracted from the Australian Snakebite Project database. Patients were categorised into three groups based on peak CK concentrations [no myotoxicity (<1000 U/L), mild (1000–10,000 U/L) and severe (>10,000 U/L)]. The odds of (mild or severe) myotoxicity was lower in patients that received early antivenom (within 6 hours post-bite) compared to those that received late or no antivenom (odd ratio was 0.186; 95% confidence interval, 0.052–0.664). A population pharmacokinetic-pharmacodynamic (PKPD) model was developed to describe the relationship between the time course of venom (a mixture of toxins) and effect (elevated CK). In addition, a kinetic-pharmacodynamic (KPD) model was developed to describe the relationship between time course of a theoretical toxin and effect. Model development and parameter estimation was performed using NONMEM v7.3. No single set of parameter values from either the PKPD or KPD models were found that could accurately describe the time course of different levels of severity of myotoxicity. The predicted theoretical toxin half-life from the KPD model was 11 ± 3.9 hours compared to the half-life of venom of 5.3 ± 0.36 hours. This indicates that the putative causative toxin’s concentration-time profile does not parallel that of venom.

**Conclusion:**

Early antivenom administration reduces the incidence of myotoxicity. The venom concentration profile does not appear to be the driver for myotoxicity following envenomation. Additional factors that affect the sensitivity of the patient to snake venom/toxins must be explored to understand the relationship with myotoxicity.

## Introduction

Snakebite envenomation remains an important public health burden in many tropical and subtropical countries, particularly those in South Asia, Southeast Asia, and sub-Saharan Africa [1]. In some cases, envenomation syndromes can have long-term effects, leading to a permanent change in quality of life or disability, such as tissue necrosis resulting in amputation, chronic kidney diseases and blindness [2]. Although snake envenomation is uncommon in Australia, its clinical implications can be life-threatening, challenging to treat and patients may require a lengthy hospital stays. Red-bellied black snakes (RBBS; *Pseudechis porphyriacus*) belong to the black snake (*Pseudechis* spp.) genus, which is one of the seven medically important territorial Australian snake genera, along with brown snakes (*Pseudonaja* spp.), tiger snakes (*Notechis* spp.), rough-scale snakes (*Tropidechis carinatus*), broad-headed snakes (*Hoplocephalus* spp.), taipans (*Oxyuranus* spp.) and death adders (*Acanthophis* spp.). The venom of *P*. *porphyriacus* contains a mixture of toxins (e.g. phospholipase A_2_, procoagulant serine protease and three-finger toxins) which are responsible for various clinical effects observed in envenomed patients [3]. The clinical envenomation syndromes of RBBS include local effects (pain and swelling), non-specific systemic symptoms (nausea, vomiting, abdominal pain, diarrhoea and headache), anticoagulant coagulopathy (elevated activated partial thromboplastin time, aPTT) and myotoxicity [4].

Local and systemic skeletal muscle injury is one of the common manifestations of snake envenomation [5]. There are a number of different myotoxins identified in Australian elapids’ venoms [6–9], although it is unclear whether all of them are clinically relevant. Phospholipase A_2_s (PLA_2_) are likely to be the major toxins responsible for myotoxicity observed in RBBS envenomed patients [9]. The binding of PLA_2_s to the plasma cell membrane of muscles can cause a disruption in membrane integrity and trigger a series of events leading to the degeneration of the muscle cells [5]. Systemic myotoxicity is the second most common systemic syndrome that occurs in Australian snake envenomation, following venom-induced consumption coagulopathy [10]. It occurs in approximately 20% of patients envenomed by RBBS. Myotoxicity can be defined as local or generalised myalgia with or without muscle weakness, in association with elevated serum creatine kinase (CK) concentration, usually defined to be greater than 1000 U/L [4]. Myotoxicity generally develops more slowly, compared to other venom effects (e.g. venom induced consumption coagulopathy), and the effect can last up to seven days post-bite. Prolonged muscle injury rarely leads to renal complications such as acute kidney injury associated with myoglobinuria and hyperkalaemia [5]. Patients that develop myotoxicity often require a longer hospital stay and more complicated treatment [11].

Creatine kinase is an enzyme that catalyses the reversible phosphorylation of creatine to phosphocreatine and the interconversion of ATP and ADP, maintaining energy homeostasis in tissues that have high or fluctuating demand for energy, such as skeletal muscles and cardiac muscle [12]. Serum CK concentration can be measured based on enzymatic activity (as U) and is often used clinically as a biomarker for damage in CK-rich tissues including skeletal muscles. Measured serum CK concentrations reflect both the natural release of CK associated with normal cellular turn-over, which yields relatively constant basal levels, as well as spikes in CK release following muscle damage [13]. Following muscle insult, serum CK concentrations begin to rise at 2 to 12 hours, peak at 1 to 3 days, and decline after 3 to 5 days [14, 15]. The delayed peak CK concentration following muscle damage is probably due the involvement of lymphatic system for transporting CK, a large molecule with molecular weight of approximately 80 kDa across the interstitial space to the vascular space [16, 17]. This process is generally slower than direct transcellular or paracellular diffusion into the bloodstream, which is common for smaller molecules [17]. The delay in abnormal CK concentration appearance may also contribute to the lack of early repeated CK testing in snakebite patients, making the early diagnosis of myotoxicity following RBBS envenomation more difficult [15]. In addition, physical activities such as strenuous exercise prior to envenomation also contribute to elevated serum CK concentrations, making diagnosis potentially more challenging [11]. Overall, serum CK concentration alone do not serve as an accurate early detector or predictor of myotoxicity.

The management of RBBS envenomation includes supportive care and antivenom therapy. In many cases, systemic envenomation syndromes following RBBS bites are mild to moderate and specific treatment or antivenom may not be required. The use of antivenom is hence, often determined by the treating clinician based on presenting signs and symptoms. For RBBS envenomation, the administration of one vial of tiger snake or black snake antivenom has shown to effectively bind and remove all free venom components [4]. The use of antivenom for RBBS envenomation is considered safe, although hypersensitivity reactions (e.g. commonly skin reactions and rarely anaphylaxis) are reported in about one quarter of patients that receive antivenom [10]. Potential benefits of antivenom administration within 6 hours after bite have been reported by Churchman et al. [4], including normalisation of aPTT and prevention of myotoxicity. While the early use of antivenom is encouraged, the decision to give antivenom to prevent myotoxicity can be complicated as patients may still present with a normal CK during the first 6 hours despite the likely occurrence of myotoxicity. Hence, an accurate risk-benefit assessment of antivenom therapy is necessary prior to the treatment to minimise patients’ exposure to unnecessary risks [10, 18].

An investigation of the relationship between time course of *P*. *porphyriacus* venom exposure (pharmacokinetic; PK) and the relationship between venom concentration and effect (pharmacodynamic; PD) is important for a better understanding of the time course of venom effect (in this case myotoxicity) following RBBS envenomation. Note the combination of pharmacokinetic and pharmacodynamic processes yields the time-course of (myo)toxicity and is termed PKPD. Knowledge of the time-course of myotoxicity following RBBS envenomation will be helpful for assisting clinicians to determine who should receive antivenom, how long patients require observation to be confident myotoxicity will not develop (given it has not developed already) and making subsequent decision for RBBS envenomation treatment.

The aims of this study are 1) to determine whether early antivenom administration (defined as within 6 hours post-bite) decreases the incidence of myotoxicity, 2) to determine whether venom (or a putative toxin) is sufficient to describe the severity of myotoxicity determined based on serum CK concentrations, and 3) to determine whether venom (which is a mixture of toxins) is an appropriate driver for myotoxicity.

## Methods

### Data

Patient data used in the present analysis were extracted from the Australian snakebite project (ASP) database, which is a collection of data of suspected or confirmed snakebites from over 200 hospitals across Australia. Details of the ASP have been described previously in a pharmacokinetic analysis of RBBS venom [19] and previous ASP studies [10]. Human research ethics committee (HREC) approval for ASP was obtained from major State and Territory HRECs, including the HREC of the Northern Territory Department of Health and Menzies School of Health Research (04/08), the Hunter New England HREC and the University of Newcastle (07/11/21/3.06), the Royal Perth Hospital Ethics Committee and South Metro Area Health Service (RA-08/003), Western Australian Country Health Service Ethics Committee (2008:03, REC200835), Tasmania Network (H00109965), Gold Coast Health Service District HREC as well as an additional ten HRECs responsible for all facilities involved. Informed consent was obtained from all enrolled patients, or from a parent or guardian for participants aged under 18 years [10].

**Fig 1:**
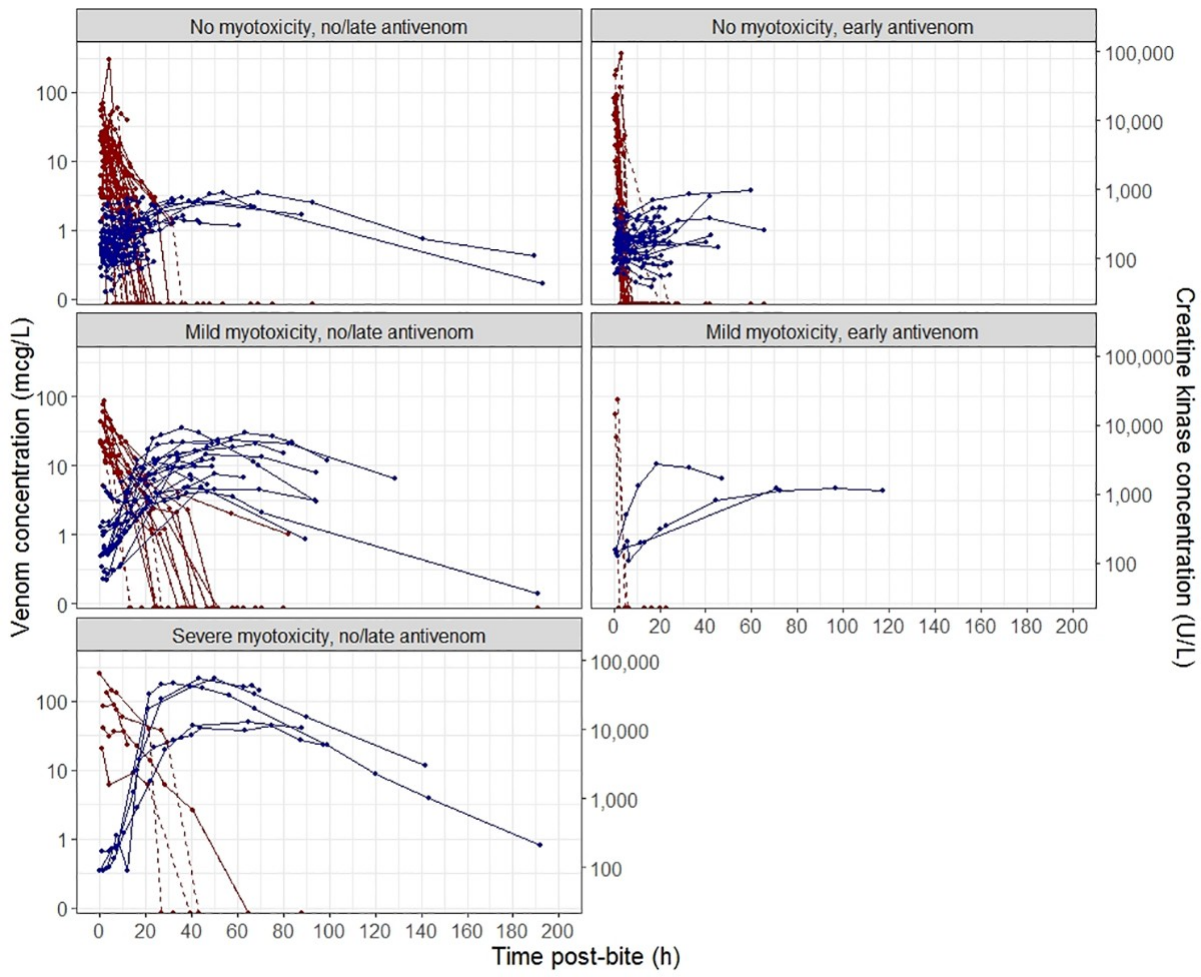
**Plots of timed venom concentration (mcg/L) [red lines] and creatine kinase (CK) concentration (U/L) [blue lines] following envenomation by red-bellied black snakes.** Solid lines are venom concentrations pre- antivenom and dashed lines are the venom concentrations post-antivenom. Patients data were categorised into three groups based on measured peak CK concentration: (a) no myotoxicity (CK < 1000 U/L); (b) mild myotoxicity (1000 U/L < CK < 10,000 U/L); and (c) severe myotoxicity (CK > 10,000 U/L). Plots on the left panel are for patients that received no or late antivenom, and on the right are those that received early antivenom (within 6 hours).

A total of 114 RBBS envenomed patients (median age 41, 2-90y; 80 male) with both timed venom concentration (pre- and post- antivenom) *and* timed CK concentration were included for the analysis from ASP between January 2002 and March 2019 (Table 1). For exploratory purposes, patients were categorised into three groups based on measured peak serum CK concentration: 1) no myotoxicity (CK < 1000 U/L); 2) mild myotoxicity (1000 U/L < CK < 10,000 U/L); and 3) severe myotoxicity (CK > 10,000 U/L). The time course of venom and CK concentrations of patients in each group are provided in Fig 1. Effective use of antivenom was determined *a priori* as a patient having received an antivenom dose within 6 hours of purported envenomation as per [4].

**Table 1:** Demographic characteristics of the study population.

	Median (range)
**Number of patients**	114
**Male:Female**	80:34
**Age (years)**	41 (2–90)
**Number of patients receiving antivenom (*n*)**	54
**Timing of antivenom (h)**	4.2 (0.67–32)
**Peak venom concentration range (mcg/L)**	20 (1.3–360)
**Measured CK concentration range (U/L)**	244 (32–56000)
• Patients with peak CK < 1000 U/L (n)	87
• Patients with 1000 U/L < peak CK < 10,000 U/L (n)	22
• Patients with peak CK > 10,000 U/L (n)	5
**Peak CK concentration measured in patients *without* myotoxicity (U/L)**	232 (48–972)
**Peak CK concentration measured in patients *with* myotoxicity (U/L)**	3019 (1065–56000)

### Model development

In this study, data were modelled within a nonlinear mixed-effects modelling framework. The population analysis was performed in NONMEM version 7.3 (ICON Development Solutions, Ellicott City, MD, USA) using the first-order conditional estimation method with interaction. Pre- and post-processing was conducted using Perl-speaks-NONMEM (PsN) version 5.22.2201, RStudio version 1.1.456 (RStudio Inc., Boston, MA, USA).

Covariates available for testing were age and sex. Decisions for model selection were based on the likelihood ratio test (LRT) with degrees of freedom equal to the number of parameters different in successive (nested) models, as well as a reduction in between subject variability (BSV) in the parameter(s) of interest, clinical relevance of the parameters and graphical goodness of fit plots. LRT requires a decrease in the objective function value (OFV) of at least 3.84 between two nested models to be statistically significant at an α-error level of 5% ([*χ*^2^], p < 0.05) for one degree of freedom.

Visual predictive checks (VPCs) was used to evaluate the final model. One thousand datasets were simulated under the final model. The 10th, 50th and 90th percentiles of the observed data were plotted and compared against their corresponding 95% confidence intervals (CI), which were calculated from the model simulations. The VPCs were also stratified based on whether the patients had received early or late/no antivenom therapy and the degree of myotoxicity to show the model performance for each group of patients.

#### The PKPD model for myotoxicity

**Fig 2:**
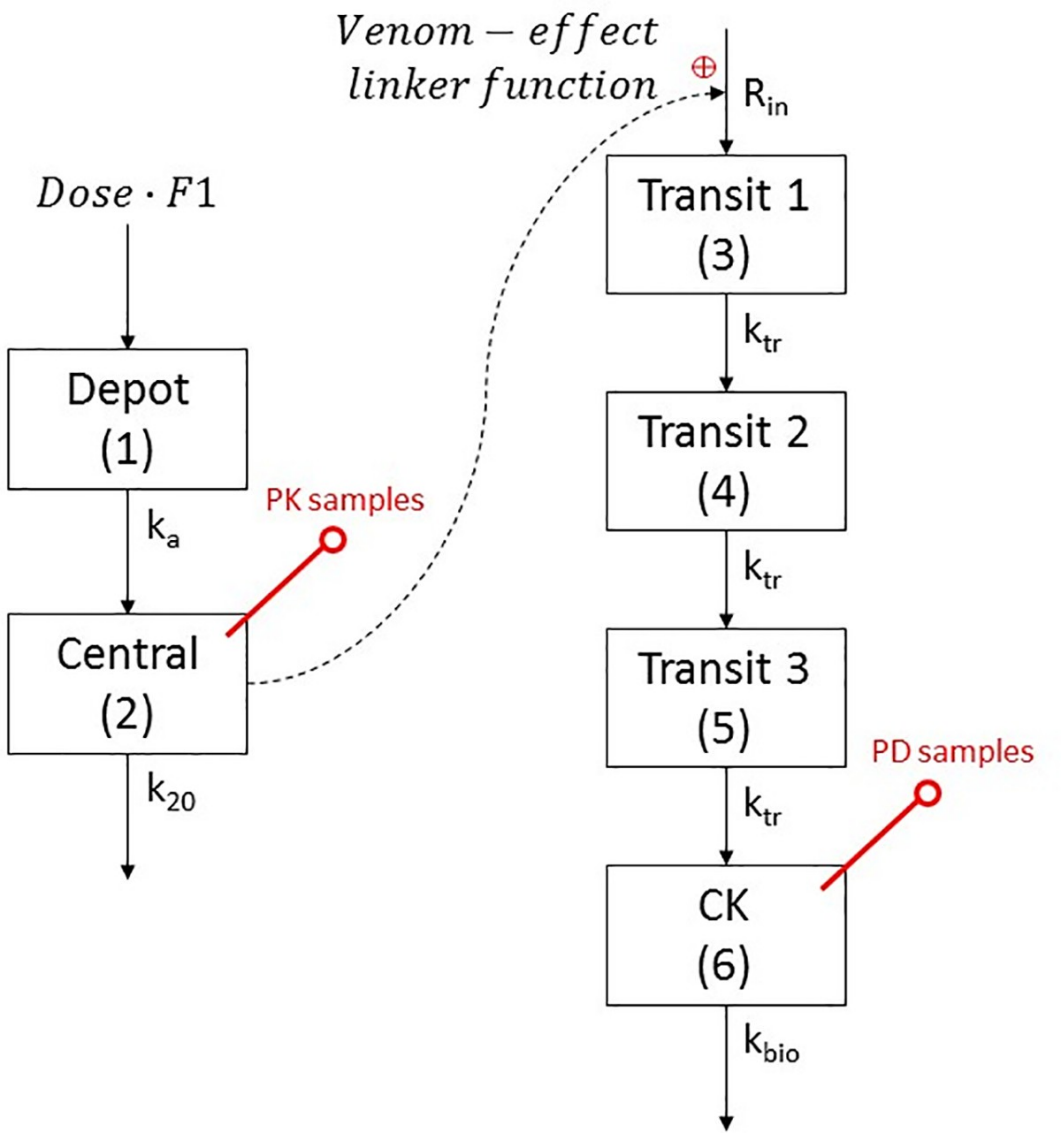
Schematic representation of the structural pharmacokinetic model for *P. porphyriacus* venom and pharmacodynamic model for creatine kinase time course. ***F*1** is the relative bioavailability; ***k***_***a***_ is absorption rate constant; ***k***_**20**_ is venom elimination rate constant; ***R***_***in***_ is a zero-order creatine kinase production rate constant; ***k***_***tr***_ is the intercompartment transit rate constant; ***k***_***bio***_ is the creatine kinase elimination rate constant. The red pins represent compartments where blood samples were collected.

The relationship between timed venom concentration and myotoxicity was investigated by a population PKPD approach. In this approach, the PK data (timed venom concentrations) and PD data (timed CK concentrations) were modelled using sequential PPP&D (*P*opulation *P*K *P*arameters and *D*ata) approach [20]. For this method, the population PK parameters obtained from previously developed population PK model for *P*. *porphyriacus* venom [19] were fixed and the PD parameters were subsequently estimated based on both PK and PD data. The details of the PK model and parameters used were described here [19]. The relationship between the plasma venom concentration and its effect (*E*_*venom*_) was described by a linker function which connected the PK data with the release of CK. Transit compartments were implemented with a turnover model as an empirical approach to describe the delayed increase in CK concentration post-envenomation that was apparent during data visualisation. A schematic of the PKPD model is shown in Fig 2.

Differential equations used to describe the amount of venom in the depot compartment (1) and the central compartment (2) were shown in Eqs 1 and 2.

$$dAdt\;(1)=-k_{a}∙A(1);A{\left(1\right)}_{t=0}=Dose∙F1$$ (1)

$$dAdt\;(2)=k_{a}∙A(1)-k_{20}∙A(2);A{\left(2\right)}_{t=0}=0$$ (2)

where *k*_*a*_ is the absorption rate constant; *k*_20_ is the venom elimination rate constant. *A* represents the amount in each compartment and *A*_*t* = 0_ is the initial condition at time zero.

Mathematically, the linker function that describes the typical effect of venom on the release of CK can be described as a function of plasma concentration, *C* and the PD parameter vector, **β** (shown in Eq 3).


$$E_{venom}=f(C(t),\beta )$$ (3)

An example linker function is shown in Eq 4, where a linear model was used as the link between venom concentration and release of CK. In this case, venom increases the release of CK from myocytes with the downstream appearance of CK into the plasma. Here the typical venom effect is a function of venom concentration in the central compartment (*C*_2_) and the slope of the directly proportional relationship between venom concentration and effect.


$$E_{venom}=\beta ∙C_{2}$$ (4)

The parameter **β** represents the slope of the relationship. The linker function is then incorporated with the parameter *R*_*in*_ (the rate of CK release from myocytes) to describe the exposure-effect relationship (Eq 5). Here venom-effect (*E*_*venom*_) stimulates the release rate of CK.

$$R_{in}={CK}_{0}∙k_{bio}∙(1+E_{venom})$$ (5)

where *CK*_0_ is the baseline amount of CK in plasma at steady state (the product of concentration and the volume of distribution of CK which is (for simplicity) assumed to be 1) and *k*_*bio*_ is the elimination rate constant of CK from the circulation. The product of *CK*_0_ and *k*_*bio*_ represents the normal zero-order release of CK into the systemic circulation (termed *R*_*in*_) in the absence of venom.

The system of equations for the turnover model with transit compartments in which venom has a stimulatory effect on CK release are shown in Eqs 6 to 10.

The differential equations for CK transit from intracellular (compartments 3 to 5) to the central circulation (compartment 6, sampling compartment for CK) are as follows:

$$dAdt\;(3)=R_{in}-k_{tr}∙A(3);\;A{\left(3\right)}_{t=0}=\frac{R_{in}}{k_{tr}}$$ (6)

$$dAdt\;(4)=k_{tr}∙A(3)-k_{tr}∙A(4);\;A{\left(4\right)}_{t=0}=A{\left(3\right)}_{t=0}$$ (7)

$$dAdt\;(5)=k_{tr}∙A(4)-k_{tr}∙A(5);\;A{\left(5\right)}_{t=0}=A{\left(3\right)}_{t=0}$$ (8)

$$dAdt\;(6)=k_{tr}∙A(5)-k_{bio}∙A(6);\;A{\left(6\right)}_{t=0}={CK}_{0}$$ (9)

where the inter-compartment transit rate constant *k*_*tr*_ was estimated from the mean transit time (MTT) and can be calculated from the following equation:

$$k_{tr}=\frac{number\;of\;transit\;compartments}{MTT}$$ (10)

#### The kinetic‐pharmacodynamic (KPD) model for myotoxicity

In addition to considering a PKPD model we also considered a kinetic-pharmacodynamic (KPD) approach in which the PK data are omitted from the PKPD analysis. The KPD analysis investigates the PD effect without the PK data which allows for imputation of the likely time course of toxin exposure. Note that the time course of changes in venom concentration (a mixture of toxins) will not necessarily parallel that of the toxin that causes the myotoxicity (e.g. PLA_2_ toxin). In the KPD model, the overall structure of the model for exposure and effect was the same as the PKPD model. However, only PD data were used for modelling and the kinetics of the concentration-driver for the effects (i.e. CK elevation) were imputed. The clearance of the toxin driver was estimated and the volume of distribution was fixed (for reasons of identifiability). Parameterisation is otherwise based on Ooi [21]. BSV for clearance and relative dose were also estimated along with other system parameters. The details of the final PKPD and KPD model are described in the S1 Text.

### Aim 1 methods—To determine whether early antivenom administration (defined as within 6 hours) decreases the incidence of myotoxicity

The odds ratio (OR) was calculated to determine the potential for a reduction in myotoxicity of administration of antivenom within 6 hours post-envenomation. A 2×2 contingency table was developed for calculating the OR from the odds of myotoxicity occurring in patients that received early antivenom therapy and the odds when antivenom was not administered within a 6-hour timeframe.

### Aim 2 methods—To determine whether venom (or a putative toxin) is sufficient to describe the severity of myotoxicity determined based on serum CK concentrations

We evaluated models for the existence of a linker function that could accurately predict the occurrence and severity of serum CK concentration based on the information on venom concentration (via a PKPD model) or a putative toxin concentration (via a KPD model). A number of models linking venom or toxin exposure to the effect observed were reviewed including: linear, log-linear, E_max_, sigmoid E_max_, quadratic, cubic, threshold and insult models. The threshold model was defined as a model where only venom concentrations above an estimated threshold concentration would result in a “venom effect”, while concentrations below the threshold will give no effect. The insult model was defined as a model in which the venom “insult”, denotes as total venom exposure (i.e. AUC), causes the muscle insult that releases CK with the assumptions that: 1) toxin causes irreversible cell injury (i.e. once the insult has occurred then subsequent venom concentration becomes irrelevant and antivenom would have no beneficial effects), and 2) after 6 hours the insult phase has completed and effect is irreversible.

### Aim 3 methods—To determine whether venom (which is a mixture of toxins) is an appropriate driver for myotoxicity

To test if venom concentration is the best driver for myotoxicity, we compare the exposure profile of venom (from the PK model [19]) to the imputed exposure profile of toxin (from the best KPD model) as drivers for myotoxicity. PK simulations for one thousand patients (without the effect of antivenom) were performed to compare the profiles of *P*. *porphyriacus* venom (mixture of toxins) and the putative toxin responsible for raised CK concentration. Simulations were performed using MATLAB version 2020a (The MathWorks, Inc., Natick, MA, USA). The median concentrations of venom (arise from PK model) and theoretical toxin (PK predicted from KPD model) and calculated half-lives were then compared. If the two profiles align this would suggest that the causative toxin PK parallels the venom PK.

## Results

### Aim 1 results—To determine whether early antivenom administration (defined as within 6 hours) decreases the incidence of myotoxicity

A 2×2 contingency table (Table 2) was developed for calculating the OR. The OR of myotoxicity occurring in patients receiving early vs late or no antivenom was 0.186 (95% confidence intervals: 0.052–0.664).

**Table 2:** Odds of myotoxicity following red-bellied black snake envenomation in patients that received early antivenom and late/no antivenom treatment.

	Myotoxicity = Y	Myotoxicity = N	Total (n)	ODDs
**Antivenom ≤ 6 h (early)**	3	35	38	0.086
**Antivenom > 6 h (late) or no antivenom**	24	52	76	0.46
**Total (n)**	27	87		

### Aim 2 results—To determine whether venom (or a putative toxin) is sufficient to describe the severity of myotoxicity determined based on serum CK concentrations

Eight different linker functions were evaluated to describe the concentration-effect relationship using both the observed PK data via a PKPD model or imputed PK data via a KPD model. A linear model provided the best fit for the venom concentration and CK concentration data. When E_max_ and sigmoid E_max_ models were evaluated, they provided similar fitting results to the linear model, and the ratio of the estimated maximum effect (E_max_) to the C_50_ (concentration that achieves 50% of peak effect) remained constant irrespective of initial starting values for parameter estimation and were the same as the value of slope in the linear model. The linear model was also not inferior to the log-linear model, quadratic model, cubic model, threshold model and insult models. The same outcomes were observed with the KPD models. The model parameters for PKPD and KPD models are shown in Table 3.

**Table 3:** Final pharmacokinetic-pharmacodynamic model and kinetic-pharmacodynamic model parameter estimates. Linear linker model was used here to describe the concentration-effect relationship.

Parameter	PKPD model parameter estimates (RSE%)	KPD model parameter estimates (RSE%)
*θ*_*CL*_ (L/h)	5.21 (FIXED)	2.21 (19)
*θ*_*V*_ (L)	39.9 (FIXED)	39.9 (FIXED)
*θ*_*F*1_	1 (FIXED)	1 (FIXED)
$\theta_{k_{a}}$ (h^-1^)	8.3 (FIXED)	8.3 (FIXED)
*θ*_*FAV*_	40 (FIXED)	40 (FIXED)
*ω*_*CL*_ (%)	47.1 (FIXED)	41.7 (42)
*ω*_*F*1_ (%)	141 (FIXED)	1166.2 (11)
*σ*_*prop* (*venom*)_ (%)	36.5 (FIXED)	-
*σ*_*add* (*venom*)_ (mcg/L)	0.258 (FIXED)	-
$\theta_{{CK}_{0}}$ (U/L)	152 (14.1)	153 (6)
*θ*_*MTT*_ (h)	34.5 (31.3)	30 (11)
$\theta_{k_{bio}}$ (h^-1^)	0.0459 (6.5)	0.0457 (7)
*θ*_*SLOPE*_	1.11 (202.7)	0.641 (36)
$\omega_{{CK}_{0}}$ (%)	69.8 (8.9)	70.1 (9)
*ω*_*MTT*_ (%)	42.1 (32.8)	50 (19)
*ω*_*SLOPE*_ (%)	572.6 (41.6)	51.1 (34)
*σ*_*prop* (*CK*)_ (%)	21.1 (8.2)	20.8 (7)
*σ*_*add* (*CK*)_ (U/L)	0.1 (299.3)	0.1 (24)

***θ***, mean value of the fixed-effect parameter; ***ω***, between-subject variability (presented as coefficient of variation percentage, CV%); ***σ***_***prop***_, proportional component of the residual variability (presented as CV%); ***σ***_***add***_, additive component of the residual variability (presented as standard deviation); ***CL***, clearance; ***V***, volume of distribution in the central compartment; ***F*1**, relative bioavailability; ***k***_***a***_, absorption rate constant; ***FAV***, factor accounting for the effect of antivenom; ***CK***_**0**_, baseline creatine kinase (CK) concentration; ***MTT***, mean transit time; ***k***_***bio***_, elimination rate constant of CK; RSE, relative standard error. Note: PK parameters were fixed to the previous population estimates for this dataset based on **[19]** as per the PPP&D method.

**Fig 3:**
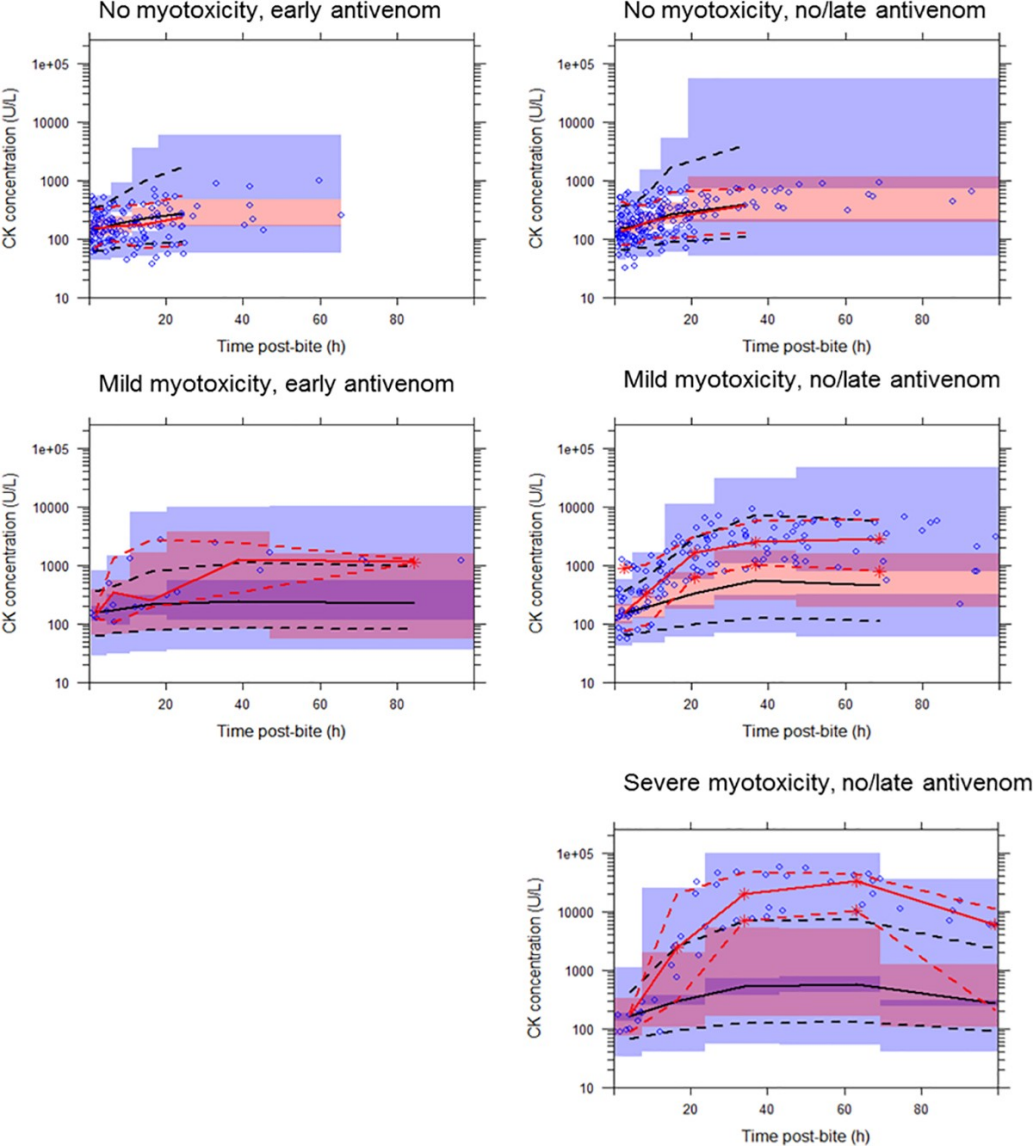
Visual predictive checks showing the observed timed creatine kinase (CK) concentration (U/L) versus time post-bite (h) described by a population pharmacokinetic-pharmacodynamic model with linear linker function to describe venom-effect relationship. The solid red line represents the median observed CK concentration, and the dashed lines represent the observed 10th and 90th percentiles. The solid black line represents the median simulated CK concentration, and the dashed lines represent the simulated 10th and 90th percentiles. Shaded areas are the corresponding 95% confidence intervals of the observed percentiles based on the simulated data. Patients data were categorised into three groups based on measured peak CK concentration: (a) no myotoxicity (CK < 1000 U/L); (b) mild myotoxicity (1000 U/L < CK < 10,000 U/L); and (c) severe myotoxicity (CK > 10,000 U/L). Plots on the left panel are for patients that received early antivenom (within 6 hours), and on the right are those that received no or late antivenom.

**Fig 4:**
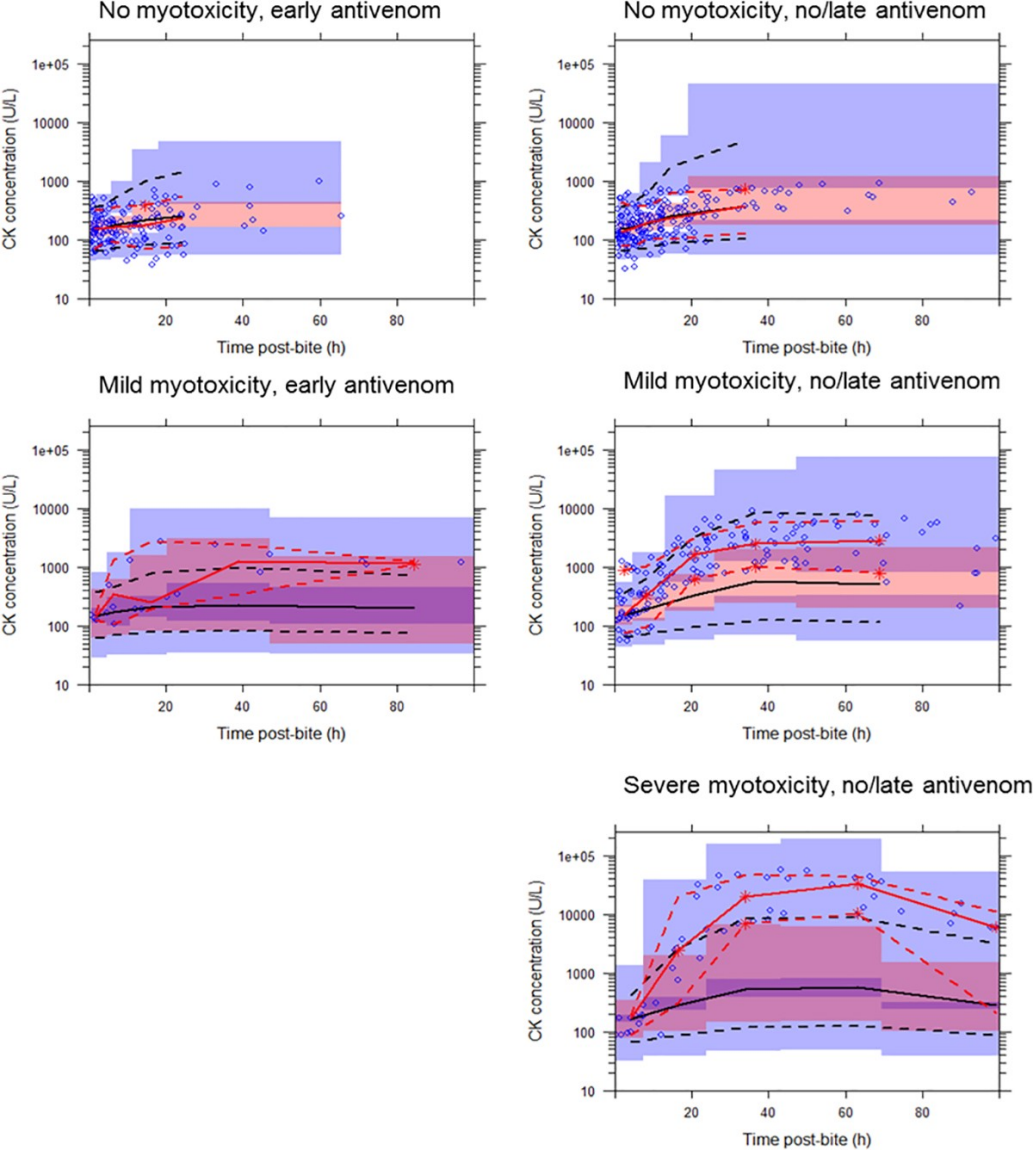
Visual predictive checks showing the observed timed creatine kinase (CK) concentration (U/L) versus time post-bite (h) described by a population kinetic-pharmacodynamic model with linear linker function to describe venom-effect relationship. The solid red line represents the median observed CK concentration, and the dashed lines represent the observed 10th and 90th percentiles. The solid black line represents the median simulated CK concentration, and the dashed lines represent the simulated 10th and 90th percentiles. Shaded areas are the corresponding 95% confidence intervals of the observed percentiles based on the simulated data. Patients data were categorised into three groups based on measured peak CK concentration: (a) no myotoxicity (CK < 1000 U/L); (b) mild myotoxicity (1000 U/L < CK < 10,000 U/L); and (c) severe myotoxicity (CK > 10,000 U/L). Plots on the left panel are for patients that received early antivenom (within 6 hours), and on the right are those that received no or late antivenom.

Both PKPD and KPD models with linear linker function were able to describe elevated CK concentrations post-envenomation well at individual levels. At the population level, both PKPD and KPD models could describe the CK concentrations well in patients with no myotoxicity and patients with mild myotoxicity who received early antivenom. However, both models under-predicted the CK concentrations in patients that received late/no antivenom and developed myotoxicity (mild and severe). This phenomenon is illustrated in the VPCs shown for the PKPD (Fig 3) and KPD model (Fig 4) with linear linker function. There was no single set of linker model parameters that enabled any of the linker functions to capture the CK concentration profile at all toxicity levels (no-myotoxicity, mild or severe myotoxicity) for either the PKPD or KPD models.

**Fig 5:**
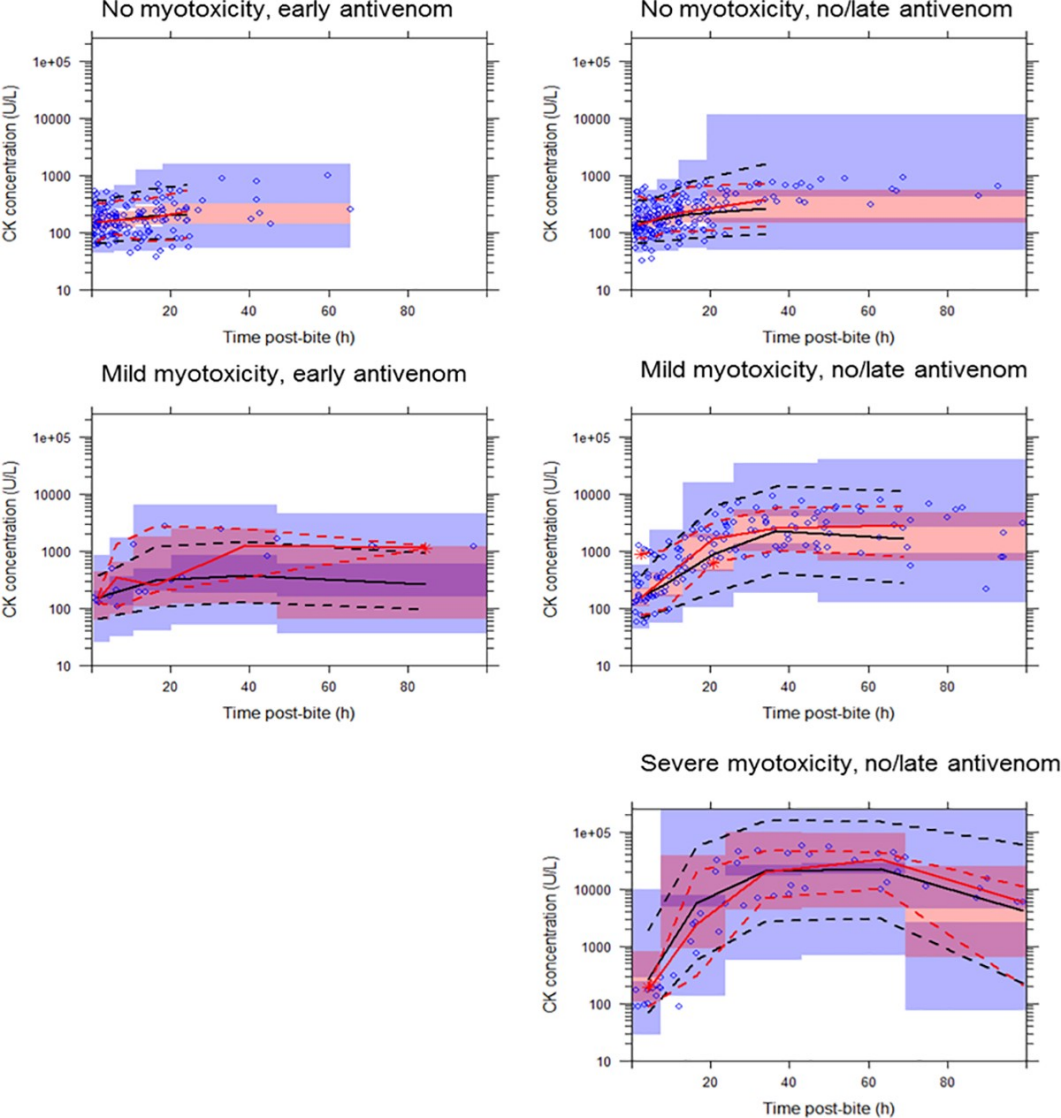
Visual predictive checks showing the observed timed creatine kinase (CK) concentration (U/L) versus time post-bite (h) described by a population kinetic-pharmacodynamic model with linear linker function (model parameters were separated for each CK group level) to describe venom-effect relationship. The solid red line represents the median observed CK concentration, and the dashed lines represent the observed 10th and 90th percentiles. The solid black line represents the median simulated CK concentration, and the dashed lines represent the simulated 10th and 90th percentiles. Shaded areas are the corresponding 95% confidence intervals of the observed percentiles based on the simulated data. Patients data were categorised into three groups based on measured peak CK concentration: (a) no myotoxicity (CK < 1000 U/L); (b) mild myotoxicity (1000 U/L < CK < 10,000 U/L); and (c) severe myotoxicity (CK > 10,000 U/L). Plots on the left panel are for patients that received early antivenom (within 6 hours), and on the right are those that received no or late antivenom.

As a diagnostic, to evaluate the linker function performance we modelled each level of CK concentration-time profiles separately. For the linear model (for example) this means a separate slope, BSV for slope and residual variability were estimated for each model at each CK level. Although this improved the performance of the PKPD model it still under-predicted the CK concentration in patients that received late/no antivenom and developed myotoxicity (mild and severe). The same diagnostic was performed for the KPD model. When the KPD model with separated model parameters for each CK level was considered it provided good predictions for the CK concentration-time profiles for all CK group levels well (Fig 5).

The diagnostic indicated that the KPD model (i.e. a model that represents a putative toxin) is able to describe the time course of CK for different levels of myotoxicity but is not able to describe the different levels of severity of myotoxicity. Models based on the venom concentration (i.e. the PKPD models) were not able to adequately describe either the CK time course or the occurrence of different myotoxicity levels.

### Aim 3 results—To determine whether venom (which is a mixture of toxins) is an appropriate driver for myotoxicity

**Fig 6:**
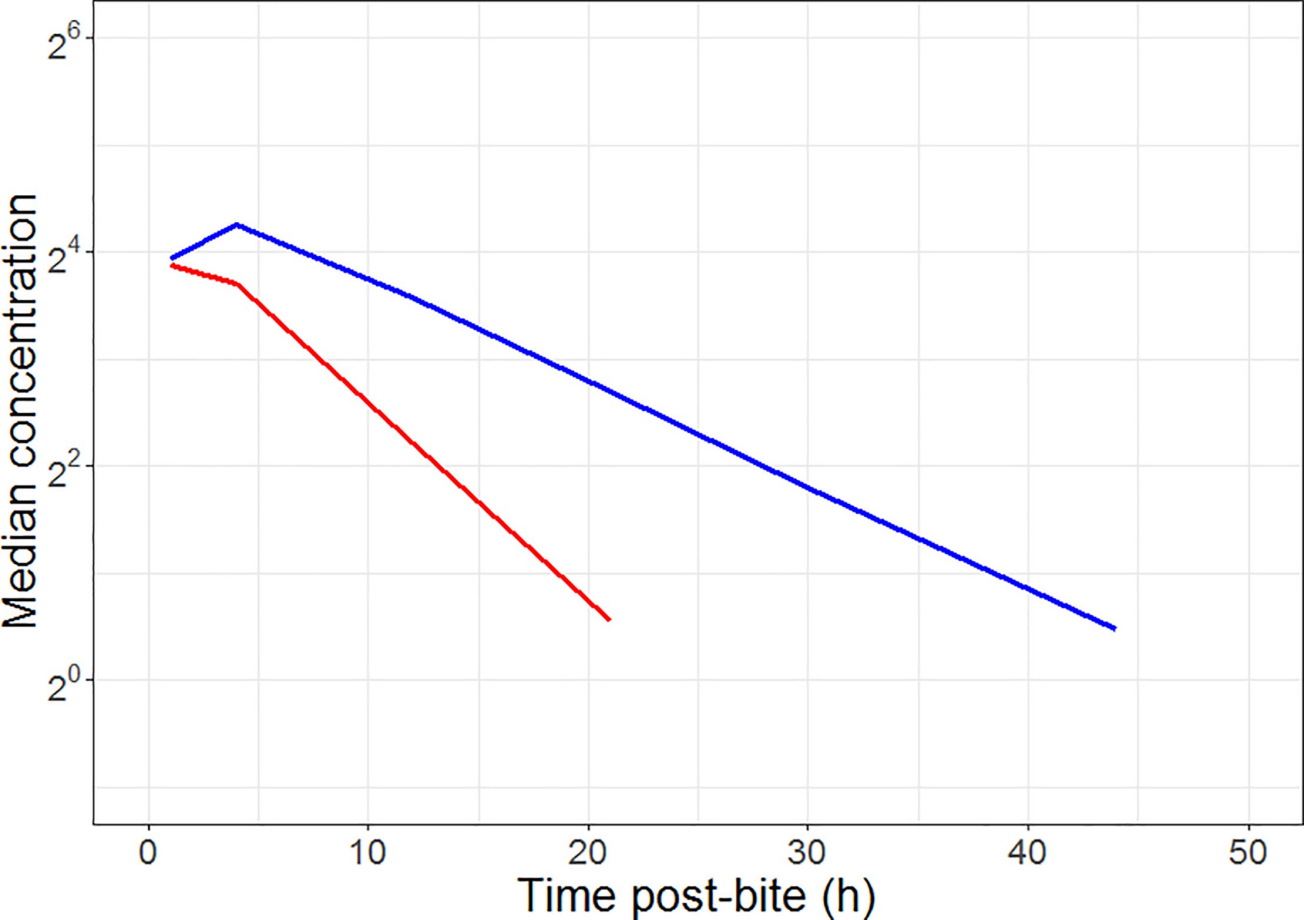
Simulation of venom profile from the population pharmacokinetic model of *P*. *porphyriacus* venom (red line) and the putative toxin profile from the final population kinetic‐pharmacodynamic model (blue line).

Plasma venom concentration-time data were simulated under the final PK model and for the imputed plasma toxin concentration-time data under the final KPD model. In all cases 1000 virtual patient profiles were simulated. Details are provided in Table 4. The median concentrations of venom (arising from the PK model) and putative toxin (PK predicted from the KPD model) are shown in Fig 6. The simulation of the imputed PK profile of toxins (driver for effect) were different from the simulated PK profile of *P*. *porphyriacus* venom. The mean half-life of *P*. *porphyriacus* venom was calculated to be 5.3 ± 0.36 hours (from the estimated clearance and volume of distribution) and the half-life of the putative toxin component of the venom was calculated to be 11 ± 3.9 hours (from the estimated clearance volume of distribution from the KPD model).

**Table 4:** Parameter estimates of the final pharmacokinetic model and kinetic-pharmacodynamic model with linear linker model (separated model parameters for each patient group) used for simulation.

Parameter	PK model parameter estimates (RSE%)	KPD model parameter estimates (RSE%)
*θ*_*CL*_ (L/h)	5.21 (6.4)	2.52 (34)
*θ*_*V*_ (L)	39.9 (12.7)	39.9 (FIXED)
*θ*_*F*1_	1 (FIXED)	1 (FIXED)
$\theta_{k_{a}}$ (h^-1^)	8.3 (FIXED)	8.3 (FIXED)
*ω*_*CL*_ (%)	47.1 (106.3)	34.4 (58.5)
*ω*_*F*1_ (%)	141 (131.7)	178.3 (23.6)

***θ***, mean value of the fixed-effect parameter; ***ω***, between-subject variability (presented as coefficient of variation percentage, CV%); ***CL***, clearance; ***V***, volume of distribution in the central compartment; ***F*1**, relative bioavailability; ***k***_***a***_, absorption rate constant; RSE, relative standard error.

## Discussion

This study investigated the occurrence and severity of myotoxicity in patients after envenomation from red-bellied black snakes and the effect of early, late or no antivenom therapy. Due to the availability of PK and PD data, we have had a unique opportunity to develop a population PKPD model and KPD model to investigate the relationship between venom or toxin exposure and the development of different degrees of myotoxicity (elevated CK concentrations). Three main inferences are gained from this work.

1^st^ inference: administration of antivenom within the first 6 hours post-bite was effective at reducing the occurrence of myotoxicity. Patients that received early antivenom therapy are 5.4 times less likely to develop myotoxicity than those that received antivenom later than 6 hours or did not receive antivenom at all. The benefits of early antivenom administrations to prevent myotoxicity has previously been reported for *P*. *porphyriacus* [4] and other Australian snake envenomation cases in human, including taipan (*Oxyuranus spp*.) [22] and mulga snake (*P*. *australis*) [23], as well as in an animal model of mulga snake (*P*. *australis*) [24]. Our result reinforces the importance of early diagnosis and treatment with antivenom (if indicated) since myotoxicity and other complications can be prevented with early administration.

**Fig 7:**
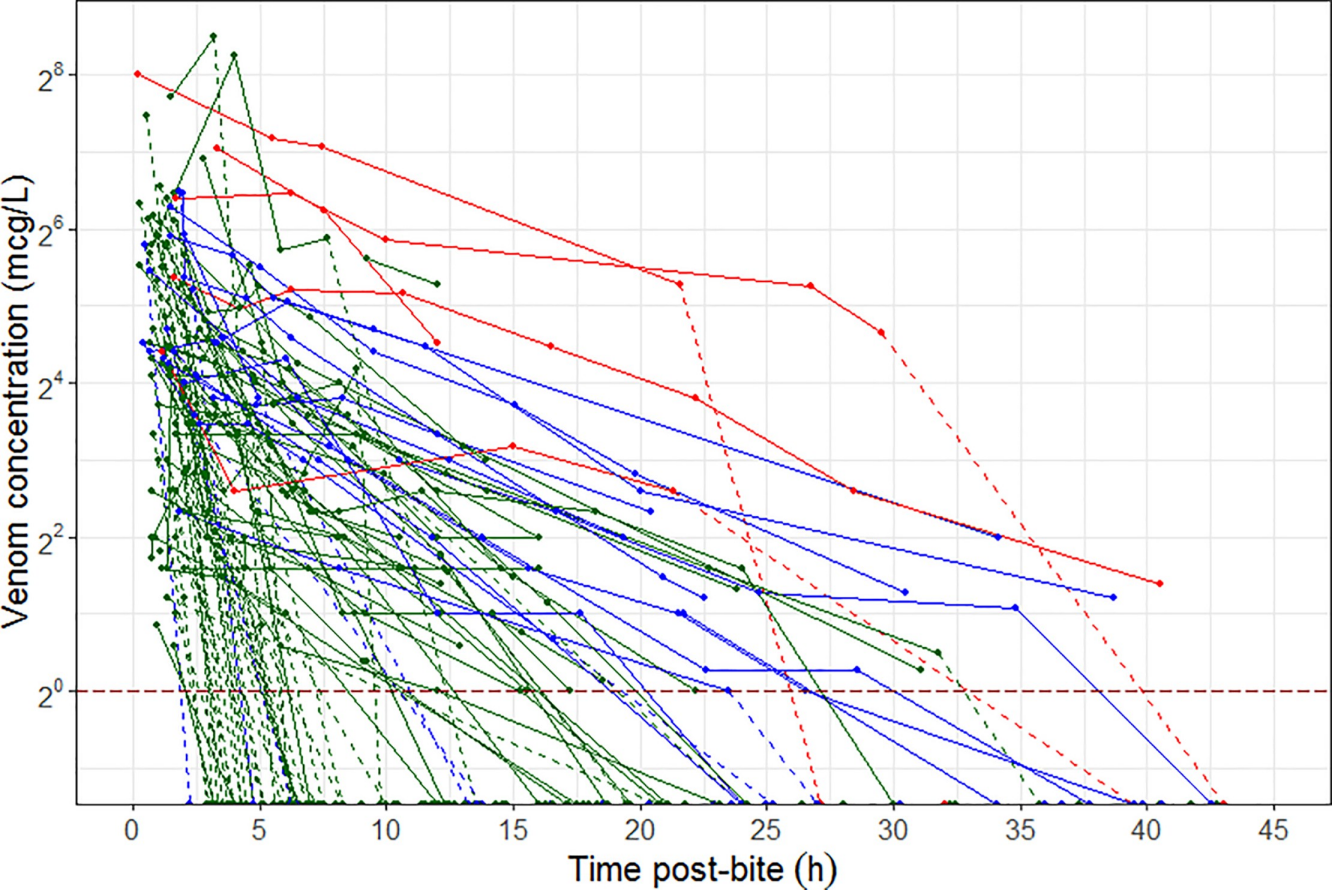
Concentration-time profile of patients that developed no myotoxicity (green), mild myotoxicity (blue), and severe myotoxicity (red). Solid lines of the profile are concentrations pre-antivenom and broken dashed lines of the profile are concentrations post-antivenom. Dark red long-dashed horizontal line is the limit of quantification for venom measurement (1 mcg/L).

2^nd^ inference: the pharmacokinetics of venom did not provide an adequate description of the occurrence or severity of elevation in CK profiles over different levels of myotoxicity. The concentration of a theoretical toxin (from KPD model) was superior to venom concentration (from PKPD model) at driving the time course of change in CK for different levels of myotoxicity. However, neither venom nor theoretical toxin concentrations were able to describe the severity of myotoxicity. The lack of relationship between venom concentration and effect is partially observable by the lack of clear visual delineation in the venom exposure for different severities of myotoxicity (Fig 7). It would be expected that more severe toxicity would be associated with higher venom concentrations. This may in part explain why the PKPD model failed to capture the different sensitivity levels of CK elevation. Of note the KPD model was also unable to predict severity of myotoxicity, but was able to describe the changes in CK concentrations well. The results suggest that a latent variable exists and contributes to the differences in snake-people sensitivity (i.e. different degree of elevated CK concentrations in patient). The latent variable may arise from: a) differences between snakes of the same species, b) different sensitivities of some people, or c) a combination of both. However, is not possible to test latent variable in a clinical study, an *in-vitro* or *in-vivo* experiments may be the way forward for testing these latent variables.

3^rd^ inference: The exposure profile of the likely (theoretical) causative toxin does not parallel the profile for venom (a mixture of all toxins). The calculated half-life of the likely causative toxin is about twice the half-life of venom (11 hours vs 5.3 hours). This suggests that the total venom concentration would not be an appropriate driver for the occurrence or severity of myotoxicity.

There are several limitations to this study many of which are inherent to clinical toxinology studies. Firstly, the dose of venom injected during envenomation was not known (or knowable). This issue has been observed in other works [19, 25]. Here, similar to previous studies, we set the dose to a nominal value (e.g. 1 mcg) and then estimate the relative bioavailability for each individual. Secondly, the exact timing of envenomation is seldom available and hence some error around the timing of toxin administration is likely as well as a the potential for substantial delays before presentation to a clinic. In this work this resulted in few early timed venom concentrations and hence the absorption phase parameters for venom were fixed. Since there was no study that report the absorption profile of *P*. *porphyriacus* venom, the absorption rate constant, *k*_*a*_ value was set to provide a similar time to peak concentration (30 minutes) reported for another Australian snake, *P*. *australis* venom following IM, SC injection in rats [24]. Furthermore, there is an uncertainty in the individual snake variability in *P*. *porphyriacus* venom composition of toxin(s) and how these vary across snakes within and between regions. Since the snake venom assay used in this study was only able to measure the total venom concentration (i.e. a mixture of toxins), this does not necessarily reflect the profile of the myotoxic component. As it is not possible to distinguish a single toxin disposition profile from the total venom concentration disposition [26], this was not able to be explored further. Of interest the PK of the likely causative toxin appeared different to that of venom. This was established using a KPD modelling approach.

Lastly, there are other possible confounders that may affect the different concentrations of plasma CK in snakebite patients. In particular, those that affect the total muscle mass may influence baseline plasma CK concentration and the amount of CK released following muscle injury. In this study, the only two variables that we were able to account for in the model were age and sex of the patient as model covariates on baseline CK. However, these did not significantly improve the overall model fit nor were they able to explain or predict the degree of severity of myotoxicity. While patients’ size (weight and height) as well as body composition may contribute to the extent of CK being released, data were not available. This could be a note to account for moving forward in the study of snakebite and myotoxicity. Another confounder to address is the administration of intravenous (IV) fluids as part of envenomation management. The administration of IV fluids may have a dilutional effect on the plasma CK concentrations and hence may have affected the peak CK observed. Unfortunately, data on the timing and the amount of IV fluids is also limited and we were not able to account for this in our final model.

Although venom concentration was not sufficient to predict the severity of myotoxicity and serum CK concentrations are too delayed, clinicians may benefit from early testing of other laboratory parameters such as aPPT and INR, which may help determine the risk of severe envenomation syndromes [15] and facilitate the decision for early antivenom use. There is a need for further work on elucidating individual toxin profiles as well as investigating potential patient sensitivity factors.

## Conclusion

This study investigates myotoxicity in patients envenomed by RBBS. We have found that early administration of antivenom reduces the occurrence of myotoxicity. Through population PKPD and KPD modelling, we have found that total venom concentration alone is not informative of the severity of myotoxicity. Additional studies are required to identify variables that may affect patient sensitivity to specific toxins.

## Supporting information

S1 Text Details of the population PKPD and KPD models. (DOCX)

## References

[1] Kasturiratne A, Wickremasinghe AR, de Silva N, Gunawardena NK, Pathmeswaran A, Premaratna R, et al. The Global Burden of Snakebite: A Literature Analysis and Modelling Based on Regional Estimates of Envenoming and Deaths. PLOS Medicine. 2008;5(11):e218. doi: 10.1371/journal.pmed.0050218 18986210 PMC2577696

[2] Waiddyanatha S, Silva A, Siribaddana S, Isbister GK. Long-term Effects of Snake Envenoming. Toxins. 2019;11(4):193. doi: 10.3390/toxins11040193 30935096 PMC6521273

[3] Fry BG. Structure–function properties of venom components from Australian elapids. Toxicon. 1999;37(1):11–32. doi: 10.1016/s0041-0101(98)00125-1 9920477

[4] Churchman A, O’Leary MA, Buckley NA, Page CB, Tankel A, Gavaghan C, et al. Clinical effects of red-bellied black snake (*Pseudechis porphyriacus*) envenoming and correlation with venom concentrations: Australian Snakebite Project (ASP-11). Medical Journal of Australia. 2010;193(11–12):696–700. doi: 10.5694/j.1326-5377.2010.tb04108.x 21143062

[5] Gutiérrez JM, Ownby CL. Skeletal muscle degeneration induced by venom phospholipases A_2_: insights into the mechanisms of local and systemic myotoxicity. Toxicon. 2003;42(8):915–31. doi: 10.1016/j.toxicon.2003.11.005 15019491

[6] Mebs D, Samejima Y. Purification, from Australian elapid venoms, and properties of phospholipases A which cause myoglobinuria in mice. Toxicon. 1980;18(4):443–54. doi: 10.1016/0041-0101(80)90052-5 7210029

[7] Dixon RW, Harris JB. Myotoxic activity of the toxic phospholipase, notexin, from the venom of the Australian tiger snake. Journal of neuropathology and experimental neurology. 1996;55(12):1230–7. doi: 10.1097/00005072-199612000-00006 8957446

[8] Harris JB, Johnson MA. Further observations on the pathological responses of rat skeletal muscle to toxins isolated from the venom of the Australian tiger snake, *Notechis scutatus scutatus*. Clinical and Experimental Pharmacology and Physiology. 1978;5(6):587–600. doi: 10.1111/j.1440-1681.1978.tb00714.x 152684

[9] Vaughan GT, Sculley TB, Tirrell R. Isolation of a hemolytic, toxic phospholipase from the venom of the Australian red-bellied black snake (*Pseudechis porphyriacus*). Toxicon. 1981;19(1):95–101. doi: 10.1016/0041-0101(81)90121-5 7222091

[10] Johnston CI, Ryan NM, Page CB, Buckley NA, Brown SG, O’Leary MA, et al. The Australian Snakebite Project, 2005–2015 (ASP-20). Medical Journal of Australia. 2017;207(3):119–25. doi: 10.5694/mja17.00094 28764620

[11] Johnston CI, Isbister GK. Australian snakebite myotoxicity (ASP-23). Clinical Toxicology. 2020:1–8. doi: 10.1080/15563650.2020.1836377 33156703

[12] Wallimann T, Wyss M, Brdiczka D, Nicolay K, Eppenberger HM. Intracellular compartmentation, structure and function of creatine kinase isoenzymes in tissues with high and fluctuating energy demands: the ‘phosphocreatine circuit’ for cellular energy homeostasis. Biochemical Journal. 1992;281(1):21–40. doi: 10.1042/bj2810021 1731757 PMC1130636

[13] Baird MF, Graham SM, Baker JS, Bickerstaff GF. Creatine-kinase- and exercise-related muscle damage implications for muscle performance and recovery. Journal of nutrition and metabolism. 2012;2012:960363. doi: 10.1155/2012/960363 22288008 PMC3263635

[14] Huerta-Alardín AL, Varon J, Marik PE. Bench-to-bedside review: Rhabdomyolysis–an overview for clinicians. Critical Care. 2005;9(2):158–69. doi: 10.1186/cc2978 15774072 PMC1175909

[15] Ireland G, Brown SGA, Buckley NA, Stormer J, Currie BJ, White J, et al. Changes in serial laboratory test results in snakebite patients: when can we safely exclude envenoming? Medical Journal of Australia. 2010;193(5):285–90. doi: 10.5694/j.1326-5377.2010.tb03909.x 20819048

[16] Lindena J, Kupper W, Friedel R, Trautschold I. Lymphatic transport of cellular enzymes from muscle into the intravascular compartment. Enzyme. 1979;24(2):120–31. doi: 10.1159/000458640 456337

[17] Sayers SP, Clarkson PM. Short-term immobilization after eccentric exercise. Part II: creatine kinase and myoglobin. Medicine and science in sports and exercise. 2003;35(5):762–8. doi: 10.1249/01.MSS.0000064933.43824.ED 12750585

[18] Isbister GK, Brown SGA, Page CB, McCoubrie DL, Greene SL, Buckley NA. Snakebite in Australia: a practical approach to diagnosis and treatment. Medical Journal of Australia. 2013;199(11):763–8. 10.5694/mja12.11172 24329653

[19] Sanhajariya S, Duffull SB, Isbister GK. Population pharmacokinetics of *Pseudechis porphyriacus* (red-bellied black snake) venom in snakebite patients. Clinical Toxicology. 2021:1–7. doi: 10.1080/15563650.2021.1896731 33832399

[20] Zhang L, Beal SL, Sheiner LB. Simultaneous vs. Sequential Analysis for Population PK/PD Data I: Best-Case Performance. Journal of Pharmacokinetics and Pharmacodynamics. 2003;30(6):387–404. doi: 10.1023/b:jopa.0000012998.04442.1f 15000421

[21] Ooi QX, Hasegawa C, Duffull SB, Wright DFB. Kinetic-pharmacodynamic model for drugs with non-linear elimination: Parameterisation matters. British Journal of Clinical Pharmacology. 2020;86(2):196–8. doi: 10.1111/bcp.14154 31729048 PMC7015753

[22] Johnston CI, Ryan NM, O’Leary MA, Brown SG, Isbister GK. Australian taipan (*Oxyuranus* spp.) envenoming: clinical effects and potential benefits of early antivenom therapy—Australian Snakebite Project (ASP-25). Clinical Toxicology. 2017;55(2):115–22. doi: 10.1080/15563650.2016.1250903 27903075

[23] Johnston CI, Brown SGA, O’Leary MA, Currie BJ, Greenberg R, Taylor M, et al. Mulga snake (*Pseudechis australis*) envenoming: A spectrum of myotoxicity, anticoagulant coagulopathy, haemolysis and the role of early antivenom therapy-Australian Snakebite Project (ASP-19). Clinical Toxicology. 2013;51(5):417–24. doi: 10.3109/15563650.2013.787535 23586640

[24] Hart AJ, Hodgson WC, O’Leary M, Isbister GK. Pharmacokinetics and pharmacodynamics of the myotoxic venom of *Pseudechis australis* (mulga snake) in the anesthetised rat. Clinical Toxicology. 2014;52(6):604–10. doi: 10.3109/15563650.2014.914526 24940643

[25] Sanhajariya S, Duffull SB, Isbister GK. Pharmacokinetics of Snake Venom. Toxins. 2018;10(2):73. doi: 10.3390/toxins10020073 29414889 PMC5848174

[26] Sanhajariya S, Isbister GK, Duffull SB. The Influence of the Different Disposition Characteristics of Snake Toxins on the Pharmacokinetics of Snake Venom. Toxins. 2020;12(3):188. doi: 10.3390/toxins12030188 32188075 PMC7150903

