# Supplementary material for: Investigating myotoxicity following Australian red-bellied black snake (Pseudechis porphyriacus) envenomation
Source: PLoS One. 2021 Sep 10;16(9):e0256653. doi: 10.1371/journal.pone.0256653 (PMC8432874; doi:10.1371/journal.pone.0256653)
Supplement: S1 Text — (DOCX) [file pone.0256653.s001.docx]

# S1 Text. Details of the population PKPD and KPD models

In this study a PPP&D sequential estimation method was utilised for fitting PK and PD data. A linear model, which is a simplest linker function, was initially utilised for describing the link between venom (or toxin) concentration and serum CK concentrations using a PKPD (and KPD) modelling approach. A turnover model with extended transit compartments (n=3) best described the delayed effect between venom or theoretical toxin concentration and CK release from myocytes to the circulation. Ternant et al. has also previously developed a similar turnover model with transit compartments to describe the serum CK and CK-MB (cardiac muscle-specific isoform of CK) concentrations in ST-segment elevation myocardial infarction patients (1). The estimated population baseline of CK was 152 U/L and 153 U/L for the PKPD model and KPD model, respectively. For both models, the half-life calculated for CK from $k_{bio}$ was 15 hours, which was similar to the half-life values reported by Ternant et al (1) of 11.2 hours and Lang et al (2) of 15 hours (for skeletal muscle-specific isoform of CK, CK-MM). Sex and age were tested as covariates on clearance and baseline CK (${CK}_{0}$). Incorporation of sex and age on venom/toxin clearance did not improve the models. The OFV reduced by 8 after incorporation of sex as covariate on ${CK}_{0}$, and the OFV further reduce by 12 after incorporation of age as covariate on ${CK}_{0}$. However, the incorporation of these covariates did not improve the BSV or the VPCs of the PKPD and KPD models and hence were not included in the final model.

## References

1. Ternant D, Ivanes F, Prunier F, Mewton N, Bejan-Angoulvant T, Paintaud G, et al. Revisiting myocardial necrosis biomarkers: assessment of the effect of conditioning therapies on infarct size by kinetic modelling. Scientific Reports. 2017;7(1):10709.

2. Lang H, Wurzburg U. Creatine kinase, an enzyme of many forms. Clinical chemistry. 1982;28(7):1439-47.
